# MWCNT-modified MXene as cost-effective efficient bifunctional catalyst for overall water splitting

**DOI:** 10.1039/d2ra00868h

**Published:** 2022-03-16

**Authors:** Syedah Afsheen Zahra, Syed Rizwan

**Affiliations:** Physics Characterization and Simulations Lab (PCSL), Department of Physics, School of Natural Sciences (SNS), National University of Sciences and Technology (NUST) Islamabad 44000 Pakistan syedrizwan@sns.nust.edu.pk syedrizwanh83@gmail.com +92 51 886 5599

## Abstract

Utilization of cost-effective, bifunctional, and efficient electrocatalysts for complete water splitting is desirable for sustainable clean hydrogen energy. In last decade, MXenes, a family of emerging two-dimensional (2D) materials with unique physiochemical properties, enticed scientists because of their use in different applications. However, insufficient electron transport, lower intrinsic chemical activity and limited active site densities are the factors inhibiting their use in electrocatalytic cells for hydrogen production. Here, we have presented material design to address this issue and introduced carbon nanotubes (CNTs) on V_2_CT_*x*_ MXene sheets for conductive network channels that enhance the ion diffusion for enhanced electrochemical activity. The SEM reveals the uniform dispersion of the MWCNTs over the MXene surface that resulted in the formation of conductive network channels and enhances reaction kinetics. The as-synthesized electrocatalyst was subjected to linear sweep voltammetry (LSV) measurements for hydrogen evolution reaction (HER) and oxygen evolution reaction (OER). The hybrid catalyst M2 exhibited an enhanced HER activity with a lower over-potential of 27 mV which is comparable to commercially available Pt-based catalysts (32 mV). Similarly, an enhanced OER activity was observed with a lower over-potential of 469 mV as compared to pristine V_2_CT_*x*_ MXene. The electrocatalyst was subjected to a durability test through chronoamperometry and was observed to be stable for 16 hours. Hence, this study opens a new avenue for future cost-effective efficient catalysts for overall water splitting as a solution to produce clean hydrogen.

## Abstract 2

Utilization of cost-effective, bifunctional, and efficient electrocatalysts for complete water splitting is desirable for sustainable clean hydrogen energy.

## Graphical abstract

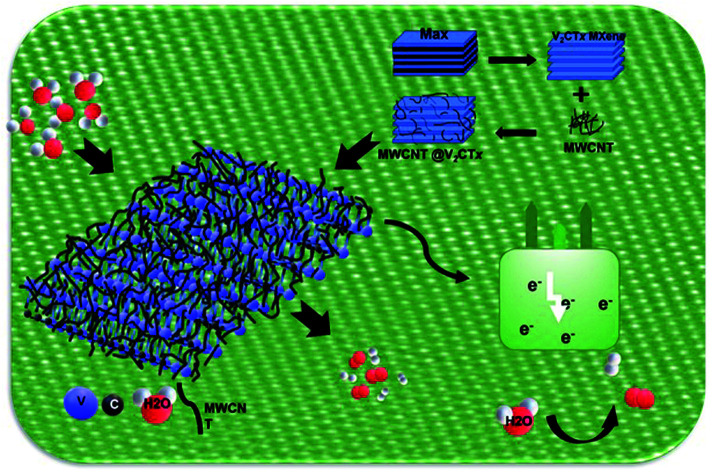

## Introduction

Global warming, increased carbon emission causing environmental hazards and shortage of renewable energy resources result in an ever growing demand for green energy production and conversion. Hydrogen fuel is considered to be the most green energy carrier to address the energy crises and environmental issues.^1–3^ Currently, hydrogen fuel is produced by the steam process *via* burning of fossil fuels. However, during this process, CO_2_ is produced causing environmental pollution.^4–9^ Electrochemical water splitting is considered as an advanced clean energy technology for hydrogen production.^10,11^ Hydrogen evolution reaction (HER) at the cathode and the oxygen evolution reaction (OER) at the anode are the two key reactions proceeding in an electrocatalytic cell for the generation of hydrogen (H_2_) and oxygen (O_2_), respectively. Precious metals (Pt, Ir, Ru) or metal oxides (Ru_2_O, IrO) are considered as efficient catalysts for electrochemical water splitting but their high cost and unavailability limit their commercialization and industrial use.^12–14^ Moreover, it is very difficult to achieve high HER and OER performance simultaneously using a single precious metal hence, there is a need to develop a cost effective, non-precious metal, bifunctional, durable catalyst for overall water splitting. In the past, transition metal chalcogenides (TMDC),^15,16^ oxides,^17–21^ phosphides,^22–27^ nitrides,^28–31^ oxy hydroxides,^32,33^ carbides^13,34^ and metal free hybrids^35,36^ have been thoroughly investigated for low-cost and effective electrocatalytic activity.

In recent years, a new family of 2D early transition metal carbides and nitrides known as MXenes have attracted much attention for their unique physical, optical, electronic, optoelectronic, thermal and magnetic properties.^37^ MXenes have general formula of M_*n*_X_*n*+1_T_*x*_ where M is early transition metal, X is carbon or nitrogen and T_*x*_ are the surface terminations like –OH, –F, –Cl or –O. This rich surface chemistry and high surface area enabled them as potential candidates for energy storage and conversion systems. MXenes are synthesized *via* wet chemical etching of A layer with hydrofluoric acid or salt soln. containing fluorine from the MAX phases. Where A is either Al or Si an element of group III A or IV A. M is early transition metal and X is carbon or nitrogen.^38^ MXenes have been widely explored for their use in lithium-ion-battery (LIBS)^39^ and supercapacitors.^40–42^ Due to the active surface functionality and chemical inertness in electrochemical potential, MXene is predicted to be a heterogeneous catalyst.^43,44^ However, very limited literature is available for the catalytic behavior of MXene. Only a few MXenes (Ti_3_C_2_T_*x*_, Mo_2_TiC_2_T_*x*_, Nb_2_CT_*x*_, V_4_C_3_T_*x*_, Mo_2_CT_*x*_) out of the largest known 2D family have been explored for catalytic activities.^45–51^ Contrary to the theoretical calculation,^43^ experimental studies reveal that Ti_3_C_2_T_*x*_ and V_4_C_3_T_*x*_ have poor HER performance with an over-potential of 600 mV (ref. 46 and 49) than Mo-based MXene (Mo_2_CT_*x*_).^46^ Therefore, many research groups are attempting to improve the catalytic performance of MXene by modification of terminal groups so that it can be used as bifunctional catalyst for overall (for both HER and OER activities) water splitting. Due to high H-binding strength (high Gibbs free energy) of oxygen, vulnerable vanadium based MXene shows a poor HER performance.^47,52^ So, the use of V_2_CT_*x*_ as an efficient catalyst is still a challenge.

Previous research shows that the introduction of the CNTs can effectively improve the conductivity of the Ti_3_C_2_T_*x*_.^57,62–67^ Their unique hollow geometry offers a high specific area that makes them suitable support for heterogeneous catalysis. One more important feature of carbon nanotube is relatively high oxidation stability due to their chemically inert nature.^67^

In this study, a facile method was adopted for the modification of V_2_CT_*x*_ MXene by introducing MWCNT for its effective use as an electrochemical catalyst. The MWCNT forms a uniform networking over the surface of the MXene sheets preventing oxidation as well as are intercalated between them that enhances the electrical conductivity and provides more active sites for the ion diffusion hence, resulting in an increased electrocatalytic activity. The modified MXene not only exhibits excellent HER activity comparable to precious metal industrial catalysts but also presented an enhanced OER hence, showed bifunctional catalyst for the overall water splitting.

## Experimental section

### Synthesis of V_2_CT_*x*_ MXene and MWCNT@V_2_CT_*x*_ MXene hybrid

**Fig. 1:**
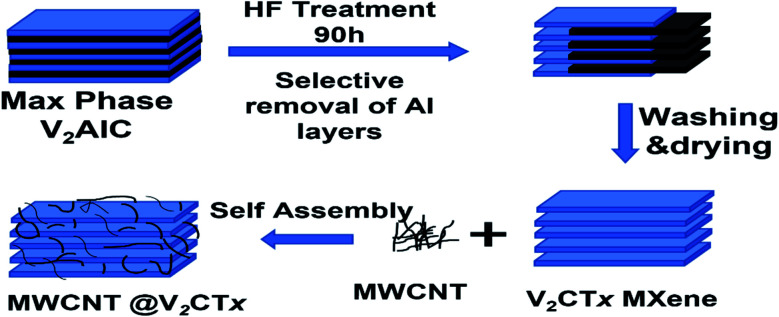
Systematic synthesis of MXene and MWCNT decorated V_2_CT_*x*_ MXene.

Fig. 1 represents the schematic for the synthesis of MXene treating MAX and MWCNT decorated MXene. The V_2_CT_*x*_ MXene was synthesized from the V_2_AlC MAX phase by wet chemical etching process. Typically, 1 g of MAX powder was added to the 15 ml of 50% Hydrofluoric Acid (HF) and kept on stirring at 200 rpm in a Teflon lined bottle for 90 hours at room-temperature. Then the mixture was centrifuged at 3500 rpm/5 min and washed several times with DI water until the PH reaches up to 6 followed by vacuum filtration to obtain multilayer (ML) V_2_CT_*x*_.

Co-precipitation route was opted for the synthesis of MWCNT/MXene hybrid. Cetyl Trimethyl Ammonium Bromide (CTAB) grafted MWCNT was prepared by dissolving 2 mg of CTAB in 20 ml DI water *via* magnetic stirring to achieve a uniform aqueous solution. After that, MWCNTs were dispersed in CTAB solution *via* probe sonication to a concentration of 0.5 mg ml^−1^. Briefly, 20 ml of CTAB grafted MWCNT solution was added dropwise to 50 ml (0.4 mg ml^−1^) of V_2_CT_*x*_ solution. The resultant mixture was probe sonicated for 10 minutes followed by vacuum filtration and vacuum drying at 50 °C overnight to get a hybrid material. In a similar fashion, four ratios 1 : 1, 1 : 2, 0.5 : 2, and 0.25 : 2 of MWCNT : MXene samples were prepared labelled as M1, M2, M3, and M4.

### Material characterization

The crystal structure and phase identification were carried out using powder X-ray diffraction (DRON-8) diffractometer equipped with Cu K-α (*λ* = 0.154 nm) source in the 2*θ* range of 3–70°. VEGA3/TESCAN 51-ADD007 scanning electron microscope (SEM) was used for the study of surface morphology. Transmission electron microscopy (TEM) analysis was carried out to further investigate the morphology. The high-resolution images were acquired on Titan 60-300 from Thermo Fischer Scientific equipped with an imaging Cs-corrector and working at 300 kV.

#### Electrochemical measurements

Gamry 1010B potentiostat workstation was used for the electrochemical testing in a three-electrode configuration using Pt wire as counter electrode and Ag/AgCl as a reference electrode in 3.5 M KCl. The working electrode was fabricated on Ni foam by dispersing 10 mg of as-synthesized MWCNT@V_2_CT_*x*_ hybrid catalyst in 500 ml DI water, 450 ml isopropyl alcohol and 50 ml Nafion (wt 5%) by ultrasonication for 35 min. Then 100 ml ink was spread on cleaned NF (1 cm × 1 cm) surface with a loading of 2–3 mg cm^−2^. The prepared electrodes were overnight vacuum-dried at room-temperature. The dried electrodes were pressed under 5–10 MPa pressure for 10 seconds. Linear Sweep Voltammetry (LSV) towards HER and OER activity was performed at constant scan rate of 10 mV s^−1^ in 1 molar KOH. All the measured potentials against Ag/AgCl were converted to RHE based on the formula of Nernst equation: *E*_RHE_ = *E*_Ag/AgCl_ + 0.059pH + 0.1976. The CV voltammograms were recorded in the voltage range of −0.4 to 0.7 V under a scan rate range of 2 mV s^−1^ to 200 mV s^−1^. The EIS was acquired at open circuit potential (OCP) in a frequency range of 20 kHz to 10 MHz using a sinusoidal signal of 10 mV. The long term durability was tested through chronoamperometry at 0.6 V.

## Results and discussions

**Fig. 2:**
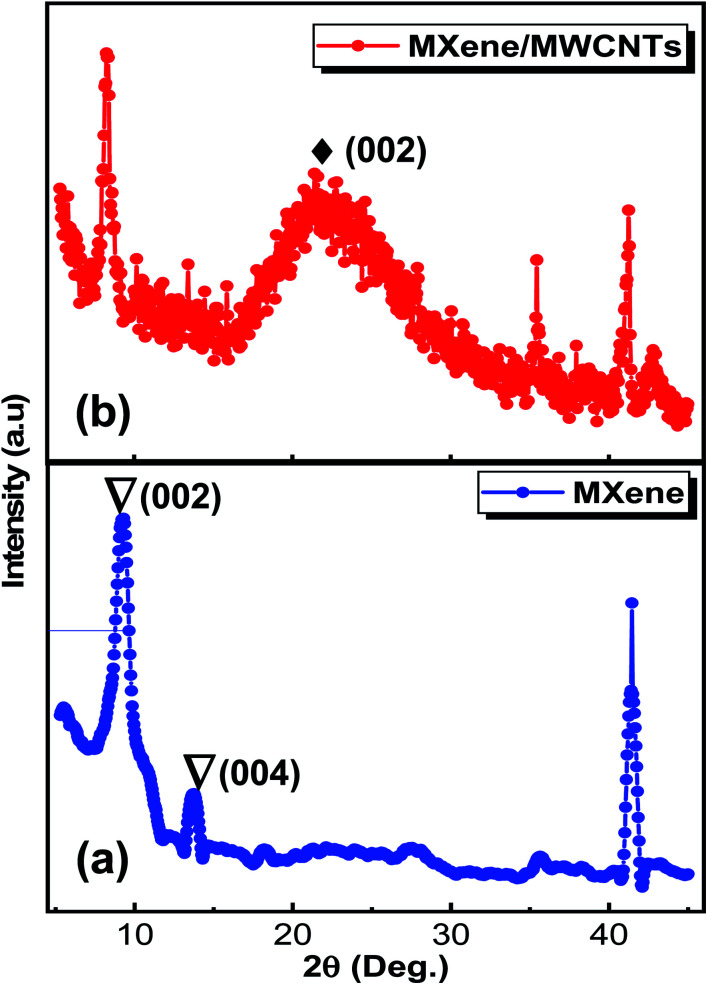
XRD patterns of (a) V_2_CT_*x*_ MXene (b) MWCNT decorated V_2_CT_*x*_ MXene.

**Fig. 3:**
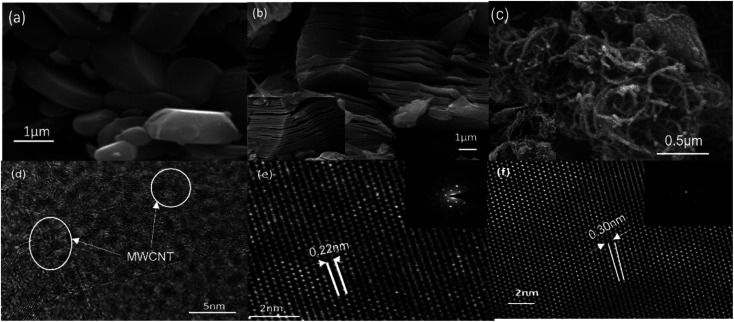
SEM images of (a) MAX phase (b) multilayer V_2_CT_*x*_ MXene (c) MWCNT@V_2_CT_*x*_ MXene. TEM images of (d) MWCNT (e) MXene (f) MWCNT@V_2_CT_*x*_ MXene.

Analysis of crystallographic structure of V_2_CT_*x*_ MXene and MWCNTs/V_2_CT_*x*_ MXene hybrid are shown in Fig. 2a and b, respectively. The most intense diffraction peak at 2*θ* = 41.3° is reduced after the HF treatment confirming the successful removal of Al layers. In addition, the shifting and broadening of (002) peak of MAX at 2*θ* = 13.4°to 2*θ* = 8.9° indicates the high *c*-axis orientation and an increased interlayer spacing (JCPDS no. 29-0101).^53^ It is obvious that the two significant peaks of MWCNTs at 2*θ* = 25.3° and 2*θ* = 42.6° (ref. 54 and 55) and the significant diffraction peaks of MXene remain intact during the hybrid fabrication, indicating its successful fabrication. The (002) plane of V_2_CT_*x*_ MXene further shifts towards a lower angle of 2*θ* = 8.24° with introduction of the carbon nanotubes. To investigate whether the MWCNT were successfully grafted on the V_2_CT_*x*_ MXene, the scanning electron microscopy (SEM) was carried out. Fig. 3a shows the SEM of MAX phase revealing the multilayer crystalline structure. While in Fig. 3b, the layers are separated forming an accordion-like structure that given an evidence of selective etching of Al after the acidic treatment.^56^ The MWCNTs forms a uniform network over the MXene sheets as can be observed from Fig. 3d; the inset represents a closer look of networking.^57^

The high-resolution transmission electron microscopy (HRTEM) is in good agreement of SEM micrographs revealing the separated basal planes of MXene (Fig. 3e). The sheets are more separated out confirming the intercalation of MWCNTs in hybrid structure (Fig. 3f). The selective area electron diffraction (SAED) (inset of Fig. 3e and f) reveals that the basal planes maintained the primitive hexagonal structure of parent MAX phase after the acidic treatment as well the hybrid formation.

### Electrochemical measurements

The electrocatalytic performance was estimated *via* HER and OER studies in 1 molar KOH using 3-electrode system. Where the as-synthesized pristine V_2_CT_*x*_ and MWCNT@V_2_CT_*x*_ catalysts were used as working electrode, platinum wire and Ag/AgCl were used as counter and reference electrode, respectively. To minimize the interference of the capacitive current *i*_R_, corrected linear sweep voltammetry was acquired at a low scan rate of 10 mV s^−1^.

### OER activity

**Fig. 4:**
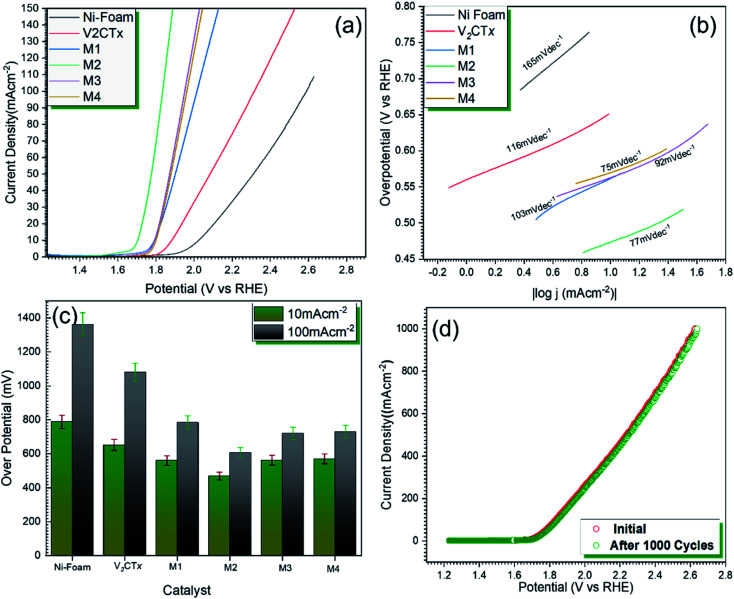
(a) OER polarization curves against RHE (b) Tafel slopes of pristine MXene and MWCNT decorated V_2_CT_*x*_ MXene (c) comparison of overpotential @ *η*_10_ and *η*_100_ mA cm^−2^ (d) durability test of MWCNT decorated MXene for 1000 CV cycles.

Fig. 4a represents the polarization curves for OER catalytic activity of V_2_CT_*x*_ MXene and MWCNT@V_2_CT_*x*_ hybrid. The hybrid material possesses lower onset potential and over-potential as compared to the pristine MXene at a current density of 10 mA cm^−2^ (*η*_10_). From Fig. 4c, it can be observed that the over-potentials for M1, M2, M3 and M4 are 560 mV, 469 mV, 562 mV and 570 mV, respectively are lower than the pristine MXene (652 mV @ *η*_10_). The higher OER activity of the hybrid is attributed to the enhanced surface area of carbon nanotubes along with the formation of uniform conductive channels for the fast ion transportation through electrode/electrolyte interface, resulting in an improved electrochemical performance. With the increasing concentration of MWCNTs, the OER activity decreases which could be attributed to the overloading and blocking of active sites. Furthermore, the Tafel plot, log(|*j*|) *versus* over-potential (*η*) was employed to verify the electrocatalytic kinetics of OER. The small Tafel slope (77 mV dec^−1^) of MWCNT@V_2_CT_*x*_ hybrid sample M2 is lower than the pristine MXene (116 mV dec^−1^) representing the synergetic effects between V_2_CT_*x*_ MXene and MWCNT (Fig. 4b).^68^ The 103 mV dec^−1^, 92 mV dec^−1^, and 75 mV dec^−1^ are the Tafel slope values for M1, M3 and M4, respectively.

The durability of the electrode is a key parameter to evaluate the performance of electrocatalyst. The catalyst was subjected to continuous cyclic voltammetry for stability test and the polarization curves were plotted after 1000 CV cycles. There was no significant change in the initial and final over-potentials after 1000 cycles confirming high durability of hybrid catalyst.

### HER activity

**Fig. 5:**
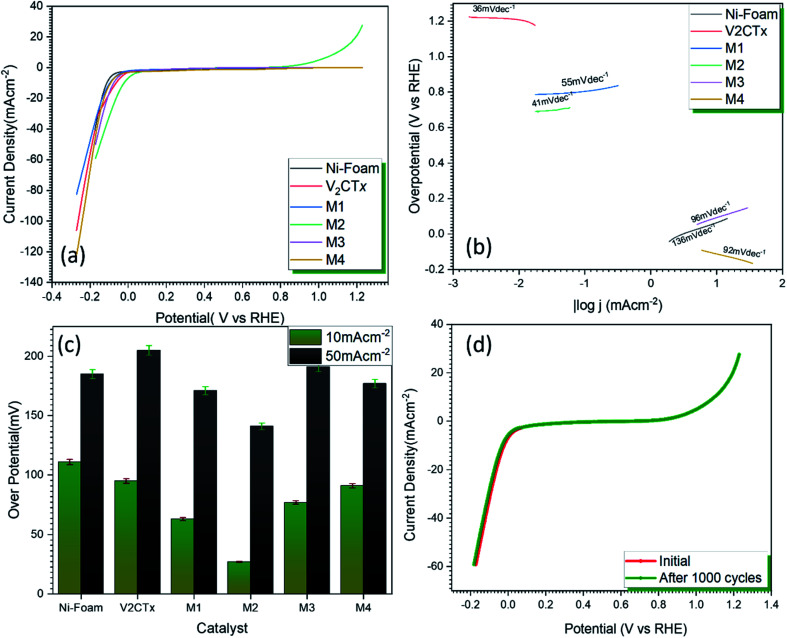
(a) HER polarization curves (b) Tafel slopes against overpotential of RHE of pristine MXene and MWCNT decorated V_2_CT_*x*_ MXene (c) comparison of over potential @ *η*_10_ and *η*_50_ mA cm^−2^ (d) durability test of MWCNT modified MXene electrode for 1000 CV cycles.

Fig. 5a represents the LSV polarization curves for HER activity of pristine V_2_CT_*x*_ MXene and MWCNT@V_2_CT_*x*_. It can be observed from the graphs that V_2_CT_*x*_ shows an average HER activity with an over-potential of 77 mV while the hybrid posses 27 mV that is comparable to commercial Pt catalysis *i.e.*, 32 mV.

The lower Tafel slope of 44 mV dec^−1^ (Fig. 5b) shows an outstanding HER kinetics obeying Volmer–Heyrovsky mechanism.

Volmer step:H_2_O + M + e^−^ → M–H_ads_ + OH^−^

Heyrovsky step:H_2_O + M–H_ads_ → H_2_ + OH^−^where, M is the active metal site, during the Volmer process in an alkaline solution M–H_ads_ intermediate is formed when hydrogen is released from the catalyst surface followed by the desorption process resulted in hydrogen gas evolution (Heyrovsky process).

This outstanding catalytic performance is the result of synergetic effects between V_2_CT_*x*_ and multiwall carbon nanotubes along with the conductive support provided by Ni foam. The porous Ni foam provides the smaller pathways for the movement of ions.^58,59^ The MWCNT forms a uniform conductive network that facilitates the ion diffusion and provide large surface area that results in the high catalytic activity. The durability is the key for the industrial scale considerations. The catalyst was subjected to 1000 cycles, and it is observed that the catalyst is stable and there is a very minor change in the catalytic activity (Fig. 5d). An over-potential of 28 mV was achieved which shows high durability of MWCNT@V_2_CT_*x*_ as shown in Fig. 5c.

### Stability test

**Fig. 6:**
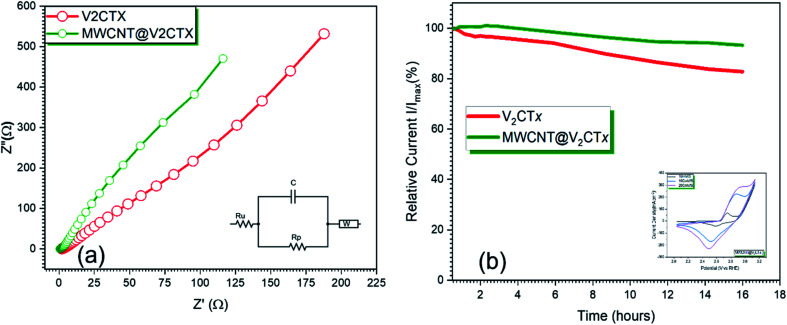
(a) EIS curves of pristine and MWCNT modified V_2_CT_*x*_ MXene with equivalent circuit model (b) chronoamperometric curves of MWCNT modified V_2_CT_*x*_ MXene for 16 h @ 0.6 V, inset: cyclic voltammograms of MWCNT decorated V_2_CT_*x*_ MXene at different scan rates.

From Fig. 6a, it can be observed from the Nyquist plots that the hybrid material has the lowest charge transfer resistance of 245.4 Ω as compared to pristine MXene (1.502 × 10^3^). This result confirms that layered V_2_CT_*x*_ MXene with high conductivity and with active sites facilitates the fast charge transfer resulting in an efficient catalytic activity because of its strong uniform interfacial linkage with MWCNTS thus, the charge transfer behavior is immensely enhanced.^60,61^ The cyclic voltammograms of the hybrid at scan rates of 10 mV s^−1^, 100 mV s^−1^ and 200 mV s^−1^ are shown in inset of Fig. 6b. It can be seen from the shape of the curve that the total capacitance is the sum of EDLC capacitance of MWCNTs and pseudo-capacitance of V_2_CT_*x*_ MXene. As MWCNT provides conductive channels for the fast ion diffusion and their increased surface are enhances the active site availability hence an increased electrochemical activity. The redox peaks are visible in the potential range of 2–3.1 V. The slight shifting of the peaks can be observed due to faradaic activity while the shape of the curve is maintained even at higher scan rates showing the symmetry and confirming the reversibility of the electrode material. The current density increases with an increase in the scan rate due to small diffusion resistance. An increase in the redox peak at higher scan rate is attributed to surface confined faradaic process that contributes to the stability of the electrode. The electron impedance spectroscopy EIS was carried out within the frequency range of 0.1 Hz to 20 kHz at a constant AC potential of 10 mV. Since, the stability of electrochemical device is a key parameter for the commercialization of the catalyst, for this purpose, the chronoamperometry was carried out at 0.6 V for 16 h which shows a very small change in current for MWCNT@V_2_CT_*x*_ confirming high stability of the catalyst.

The EIS data was subject to Randle's model fitting with additional Warburg element for fitting purposes. Table 1 shows a comprehensive analysis for the different components of equivalent circuit confirming the low charge transfer resistance of MWCNT modified V_2_CT_*x*_*i.e.* 245.4 Ω as compared to pristine MXene with 1.502 × 10^3^ Ω thus responsible for fast kinetics of the reaction.

**Table 1:** Fitting parameter values of the EIS data of V_2_CT_*x*_ MXene and MWCNT@V_2_CT_*x*_

Sample	*R*_s_ (Ω)	*R*_ct_ (Ω)	*C* (F)	*W* (Ω s^−(1/2)^)
V_2_CT_*x*_	3.30	1.502 × 10^3^	4.6 × 10^−3^	16.08 × 10^−3^
MWCNT@V_2_CT_*x*_	1.6	245.4	10.74 × 10^−3^	41.59 × 10^−3^

A comparison of this research study to MXene-based electrocatalysts is given in Table 2.

**Table 2:** Summary of electrocatalytic (HER & OER) activities of MXene-based electrocatalysts

Catalyst	Electrolyte	Activity	Over-potential @ 10 mA cm^−2^ (*η*_10_) (mV)	References
VS_2_/V_2_CT_*x*_	1 M KOH	HER	164	66
MWCNT/V_2_CT_*x*_	1 M KOH	HER	27	This work
Co^3+^–V_2_CT_*x*_	1 M KOH	OER	420	69
MWCNT/V_2_CT_*x*_	1 M KOH	OER	469	This work
Co/N-CNTs@Ti_3_C_2_T_*x*_	0.1 M KOH	OER	411	65
V_4_C_3_T_*x*_	0.5 M H_2_SO_4_	HER	200	47
Mo_2_CT_*x*_	1 M H_2_SO_4_	HER	230	70
Ti_3_C_2_T_*x*_ flakes	0.5 M H_2_SO_4_	HER	385	71
Co^3+^–Ti_2_CT_*x*_	1 M KOH	OER	420	69
Co^3+^–Cr_2_CT_*x*_	1 M KOH	OER	420	69
MoS_2_/Ti_3_C_2_-MXene@C	0.5 M H_2_SO_4_	HER	135	72
Co–B_i_/Ti_3_C_2_T_*x*_	1 M KOH	HER	250	73
FeNi-LDH/Ti_3_C_2_T_*x*_	1 M KOH	OER	298	74
N–Ti_2_CT_*x*_	0.5 M H_2_SO_4_	HER	215	75
NiS_2_/V_2_CT_*x*_	1 M KOH	HER	179	76
CoNi-ZIF-67@Ti_3_C_2_T_*x*_	1 M KOH	OER	323	77
MoS_2_⊥Ti_3_C_2_	0.5 M H_2_SO_4_	HER	110	78
Co–MoS_2_@Mo_2_CT_*x*_	1 M KOH	HER	112	79
Ti_3_C_2_T_*x*_ nanofibers	0.5 M H_2_SO_4_	HER	169	71

## Summary

In summary, a novel MWCNT–MXene heterostructure was constructed for efficient bifunctional electrochemical catalyst for an overall water splitting. For comparison, pristine MXene catalyst was also synthesized and characterized. MWCNT@V_2_CT_*x*_ showed a remarkable HER activity and an OER performance superior to pristine MXene. This enhanced performance may be attributed to the high conductivity of MXene sheets holding many active sites and uniform networking of MWCNTS over the surface, enhancing charge transfer ability and aggregation prevention. This work reveals that MWCNT@V_2_CT_*x*_ has a potential to replace commercial noble metal electrocatalysts. Moreover, this study opens the doors for other construction of MWCNT hybrids of MXene based materials for efficient bifunctional electrocatalysis for an overall water splitting.

## Author contributions

Syedah Afsheen Zahra performed experimentation and paper writing; and Syed Rizwan conceived the research concept, helped in paper writing, and supervised the complete project.

## Conflicts of interest

There are no conflicts to declare.

## Supplementary Material
